# A Robust Framework for Data Generative and Heart Disease Prediction Based on Efficient Deep Learning Models

**DOI:** 10.3390/diagnostics12122899

**Published:** 2022-11-22

**Authors:** Raniya R. Sarra, Ahmed M. Dinar, Mazin Abed Mohammed, Mohd Khanapi Abd Ghani, Marwan Ali Albahar

**Affiliations:** 1 Computer Engineering Department, University of Technology, Baghdad 00964, Iraq; 2 College of Computer Science and Information Technology, University of Anbar, Ramadi 31001, Iraq; 3 Biomedical Computing and Engineering Technologies (BIOCORE) Applied Research Group, Faculty of Information and Communication Technology, Universiti Teknikal Malaysia Melaka, Durian Tunggal 76100, Malaysia; 4 Department of Computer Science, Umm Al Qura University, Mecca 24211, Saudi Arabia

**Keywords:** heart disease prediction, artificial intelligence, deep learning, generative adversarial network, data augmentation, one-dimensional convolutional neural network, bi-directional long short-term memory

## Abstract

Biomarkers including fasting blood sugar, heart rate, electrocardiogram (ECG), blood pressure, etc. are essential in the heart disease (HD) diagnosing. Using wearable sensors, these measures are collected and applied as inputs to a deep learning (DL) model for HD diagnosis. However, it is observed that model accuracy weakens when the data gathered are scarce or imbalanced. Therefore, this work proposes two DL-based frameworks, GAN-1D-CNN, and GAN-Bi-LSTM. These frameworks contain: (1) a generative adversarial network (GAN) and (2) a one-dimensional convolutional neural network (1D-CNN) or bi-directional long short-term memory (Bi-LSTM). The GAN model is utilized to augment the small and imbalanced dataset, which is the Cleveland dataset. The 1D-CNN and Bi-LSTM models are then trained using the enlarged dataset to diagnose HD. Unlike previous works, the proposed frameworks increase the dataset first to avoid the prediction bias caused by the limited data. The GAN-1D-CNN achieved 99.1% accuracy, specificity, sensitivity, F1-score, and 100% area under the curve (AUC). Similarly, the GAN-Bi-LSTM obtained 99.3% accuracy, 99.2% specificity, 99.3% sensitivity, 99.2% F1-score, and 100% AUC. Furthermore, time complexity of proposed frameworks is investigated with and without principal component analysis (PCA). The PCA method reduced prediction times for 61 samples using GAN-1D-CNN and GAN-Bi-LSTM to 68.8 and 74.8 ms, respectively. These results show that it is reliable to use our frameworks for augmenting limited data and predicting heart disease.

## 1. Introduction

The terms “heart diseases” (HD) and “cardiovascular diseases” (CVDs) are used interchangeably in the literature. They refer to different disorders that affect the blood vessel system and the heart’s functionality. Some of these disorders are present at birth, including congenital heart diseases (CHD). Other heart disorders, such as rheumatic heart disease (RHD), coronary artery disease (CAD), arrhythmia (ARR), valvular heart disease (VHD), congestive heart failure (CHF), and atherosclerosis develop later in life [1,2]. According to the World Health Organization (WHO), 17.7 million people die annually from CVDs. By 2030, the number of annual deaths caused by CVD is expected to rise to over 23 million [3].

CVDs have several risk factors. The WHO has identified three broad categories of risk factors: changeable, non-changeable, and novel. Changeable risk factors are those that can be modified, such as high cholesterol level, high blood pressure, lack of physical activity, smoking, high consumption of saturated fat, obesity, a diet high in processed food and low in fruits and vegetables, depression, mental stress, and use of some medications. Non-changeable factors are those that cannot be modified, such as gender, age, family history, genetics, and racial background. Novel risk factors are those that are completely new. An increased amount of homocysteine in the blood and abnormal thickening of the blood are examples of novel risk factors [4,5]. A diagnosis of heart disease refers to the process of detecting the presence of a cardiac-related health issue and the degree to which it has progressed. HD can be detected properly from analyzing (1) risk factors; (2) patient physiological signals, such as heart rate, body temperature, blood pressure, etc.; (3) patient symptoms, such as anxiety, chest pain (angina), shortness of breath, nausea, irregular heartbeat (palpitations), dizziness, fatigue, etc.; and (4) clinical tests, such as blood tests, electrocardiograms (EKG/ECG), echocardiograms (Echo), computed tomography (CT) scans, chest X-rays, and cardiac magnetic resonance imaging scans (MRI) [6,7,8]. Medical professionals rely on their own clinical expertise when examining these multiple parameters about a patient and form a diagnosis. However, this manual approach to HD diagnosis might result in errors. Furthermore, as cardiovascular technology advances, it becomes more capable of recording huge amount of data, adding to the already heavy workloads of medical professionals [9,10]. Therefore, it became more challenging and time-consuming for doctors to correctly detect heart disease. Therefore, many ML and DL methods have been employed to detect cardiac disease automatically with less involvement from doctors. ML is the subfield of artificial intelligence (AI) that refers to the process of training a computer program to acquire different features of a given set of data and make decisions like a human does [6,11]. ML approaches have evolved as a more effective means of assisting the healthcare sector in the diagnosis of CVD, and they have been employed in the literature for CVD prediction and classification [12,13]. However, ML methods still require the manual use of engineering techniques for feature extraction, feature selection, and feature reduction [14]. Therefore, the major drawbacks of such techniques are to identify and use the appropriate features from the patient data. Recently, DL methods have proven themselves as a more accurate and effective method for the prediction of heart diseases as compared to conventional ML methods [15]. DL-based models eliminate the need for a domain expert for feature engineering techniques since they can automatically extract high-level features from raw input data [16]. Despite the advantages of DL models over classical ML methods, they demand computationally intensive training with a large dataset [17]. Consequently, the results of deep learning classifiers can be improved significantly if a vast dataset is available [18]. The main aim of this work is to propose a DL framework for the generation of data as well as the prediction of heart disease. The following list summarizes the most important contributions of this work:

1. Propose two deep learning (DL) models based on one-dimensional convolutional neural network (1D-CNN) and bidirectional long short-term memory (Bi-LSTM) for HD diagnosis.
2. Propose a generative adversarial network (GAN) model to augment the imbalanced and limited dataset to have a balanced distribution and larger dataset for training the predictive models (1D-CNN, and Bi-LSTM) and improving their performance.
3. Reduce model complexity, computation time and dataset dimensionality for more quick diagnosis using a fine-tuning and dimension reduction technique.
4. Evaluate the effectiveness of the proposed DL models using various performance measures and compare with conventional ML and DL models such as support vector machines (SVM) and artificial neural networks (ANNs).

The rest of this paper is as follows. In Section 2, earlier works related to the use of DL models for the purpose of detecting and predicting HD are presented. Section 3 presents the proposed methodology in detail, including the data collection and pre-processing, the GAN model for data augmentation, the dimensionality reduction technique, the 1D-CNN and Bi-LSTM models for HD prediction, and a brief overview of the SVM and ANN models that have been used for result comparison. Section 4 discusses the experimental results and provides a comparison with the state-of-the-art methods. In Section 5, a conclusion is presented.

## 2. Related Work

Most works that have been done in the past simply employ an artificial neural network (ANNs) or deep neural network (DNNs) to predict cardiac diseases. Using the Cleveland dataset that is in the machine learning repository at the University of California, Irvine, Ali et al. [19] proposed an automatic diagnostic framework for the identification of heart disorders. They used a statistical model named χ^2^ to get rid of features that weren’t necessary, and they classified the data using Deep Neural Network (DNN). They demonstrated that DNN performs better than ANN on small datasets by removing irrelevant features and comparing the two methods’ results. Their model achieved an accuracy rate of 93.33%. However, they did not demonstrate the time-consuming aspect of the model that was proposed. Das et al. [20] used a variety of ML and DL classification methods to carry out their research for a comparative analysis. They achieved the maximum accuracy of 92% for ANN, demonstrating that deep learning models are superior to other approaches when it comes to the classification of HD. Mienye et al. [21] suggest using an upgraded sparse autoencoder based artificial neural network (ANN) to predict cardiac disease. The sparse autoencoder was utilized to learn the most accurate representation of the data, while the artificial neural network was used to create predictions based on the records that were learned. The accuracy of the ANN classifier increased because of the technique that was proposed, and it eventually reached 90%.

The UCI Machine Learning Heart Disease dataset was also used by Bharti et al. [18] to test and compare a variety of ML and DL methods for the purpose of predicting heart disease. They concluded that ML algorithms performed better in situations where the dataset was not particularly large. The amount of time needed to perform computations was cut down, and an accuracy of 94.2% was achieved by using the deep learning approach. The fact that the dataset was not very large was one of the limitations, which meant that the findings of the deep learning method were restricted.

In recent years, more advanced forms of DL, including convolutional neural networks (CNNs), and long short-term memory (LSTM) have been employed for the purpose of HD identification. The authors Sajja et al. [22] created a model for the prediction of HD that was based on convolutional neural networks (CNNs). In contrast to the more conventional machine learning (ML) approaches, the newly presented model was able to attain an accuracy of 94.78% on the UCI-ML Cleveland dataset. One of the limitations was, however, that the model complexity was increased, which increased the time complexity of the model that they proposed.

An improved Recurrent Neural Network (RNN) model of deep learning was proposed by Krishnan et al. [23] for improving the accuracy of HD prediction. The existence of numerous Gated Recurrent Units (GRU) has improved the performance of the RNN model, which now has an accuracy rate of 98.4% and a processing time that is much faster. However, research needs to be done utilizing larger datasets to prove that the proposed deep learning model is accurate. OCI-DBN is an ideally configured and upgraded deep belief network that was offered by Ali et al. [24] for the purpose of predicting cardiac disease. They came up with the idea of using a stacked genetic algorithm (SGA) to produce the best possible DBN configuration. A classification accuracy of 94.61% was achieved by the suggested OCI-DBN, which resulted in an improvement in the prediction of heart disease.

The literature on HD diagnosis shows that many researchers have used advanced DL methods to enhance prediction precision. Yet, model and computation complexity receive comparatively less attention. Furthermore, the data that went into the models came from a small and unbalanced set, which made the models’ predictions less accurate. For establishing better and more reliable diagnostic performance, it is necessary to address these research gaps associated with HD diagnosis. Therefore, the GAN-1D-CNN and GAN-Bi-LSTM diagnostic frameworks are presented as a potential solution in this study. In contrast to other papers, we employed a GAN model to produce virtual data that are identical to the available data to address the issue of limited and imbalanced data. We then used 1D-CNN, Bi-LSTM, and two DL models for HD prediction. These models are fine-tuned using a fine-tuning technique to find the most efficient and simplest architecture.

## 3. Materials and Methods

**Figure 1:**
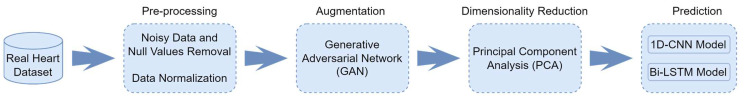
Basic process of GAN-1D-CNN and GAN-Bi-LSTM frameworks.

In this study, GAN-1D-CNN and GAN-Bi-LSTM frameworks have been developed to predict the presence of HD. These frameworks include two main stages. The first stage augments the available dataset using the generative adversarial network (GAN) model, while the second stage uses an HD predictive model based on either 1D-CNN or Bi-LSTM. Figure 1 depicts the basic process of the proposed frameworks.

The following sub-sections detail the methodologies and algorithms of the GAN-1D-CNN and GAN-Bi-LSTM frameworks. Section 3.1 explains the details of the dataset used to train and test these frameworks. It also describes the data preprocessing phase, which includes handling null values, removal of noise, and data normalization. Section 3.2 explains the proposed generative adversarial network (GAN) model for dataset augmentation. Section 3.3 describes the dimensionality reduction technique used in this study. Section 3.4 deals with the proposed 1D-CNN and Bi-LSTM models for HD detection. Section 3.5 provides an outline of the SVM and ANN models.

### 3.1. Data Sources and Pre-Processing

For this work, the HD data related to using sensors is collected from the publicly available University of California, Irvine (UCI) ML repository. Two datasets are collected: the Cleveland and Statlog datasets [25,26]. These two datasets are the most commonly utilized datasets in HD prediction [19]. The Cleveland dataset is comprised of 303 records and 14 attributes. A total of thirteen (13) attributes including age, gender, type of chest pain, blood pressure at rest, cholesterol level, maximum heart rate, fasting blood glucose, angina during exercise, resting ECG, ST depression, ST slope, thalassemia, and number of fluoroscopically colored vessels are considered as input attributes. The presence or absence of cardiac disease is determined by a final output attribute with binary values of 0 and 1 to show whether a person has an HD [27]. These attributes represent the vital signs of the human body, which are collected using various sensors such as a pulse sensor, and a blood pressure sensor. The Statlog dataset similarly includes 14 features, however it has 270 records. Both datasets (i.e., Cleveland and Statlog) have the same type and number of features. Table 1 describes the attributes of the two datasets in further detail. Given the relatively small size of these two datasets, a larger public HD dataset (Comprehensive) [28] was also collected for further experimentation and results comparison. the Comprehensive dataset combines several different datasets. These include the Cleveland (303 records), Hungarian (294 records), Switzerland (123 records), Long Beach VA (200 records), and Statlog (270 records). In total, there are 1190 records and 12 features in the Comprehensive dataset. These 12 features are all the same features of the Cleveland and Statlog datasets, but with two features being dropped, which are thalassemia and the number of significant vessels colored by fluoroscopy.

Considering the collected datasets, there were some absent or undesirable variables which had the potential to make the overall prediction of HD less accurate. As a result, these values were removed. Furthermore, some features such as age, blood pressure, max heart rate, and cholesterol level had uneven data distribution, which, if not properly managed, might lead to incorrect results in predictive model training. Therefore, a standard feature scaling approach was utilized during the pre-processing stage to achieve a normal distribution of the data. This method examines each data point ($x_{i}$) and computes the new standardized data point, which is denoted by ($x_{\mathrm{std}}$), by subtracting the mean (μ) and dividing by the standard deviation (σ), as demonstrated in Equation (1) [22].

(1) $$\begin{array}{c} x_{\mathrm{std}}=\frac{x_{i}-\mu }{\sigma }\; \end{array}$$

### 3.2. Data Augmentation Model

**Figure 2:**
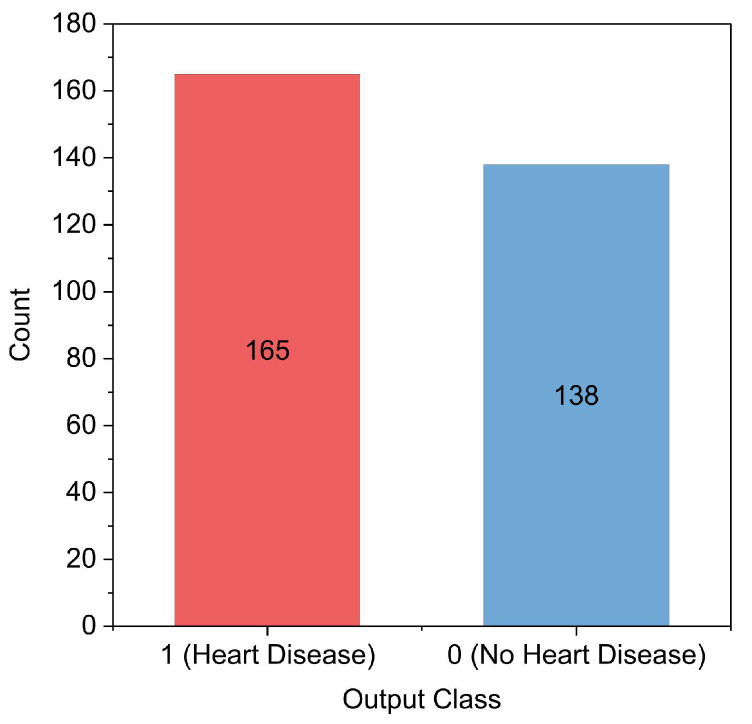
Cleveland dataset target classes.

The collected datasets (i.e., Cleveland and Statlog) are imbalanced, as the target output distribution is not equal. As can be observed in Figure 2, there are 165 people in the Cleveland dataset who have HD, and 138 people who do not. The same issue is observed in the Statlog dataset. Furthermore, both datasets contain a relatively small number of samples (i.e., 303 records for the Cleveland dataset and 270 records for the Statlog dataset). Having a dataset that is either limited or imbalanced can cause an overfitted model, which then results in an insufficient level of prediction accuracy when evaluated for new data [29,30]. Overfitting is a problem where models perform well in training but poorly when tested on new unseen data [31,32]. To address this issue, we modeled a generative adversarial network (GAN) that can produce more data from the collected datasets. The GAN is essentially a two-network system that consists of a generator and a discriminator. These two networks simultaneously compete against each other and collaborate with each other. After a significant amount of training iterations, the generator network can produce fake data that looks like real data, and the discriminator network recognizes this data as real [33]. After the fake data have been produced, they can be utilized alongside the actual data for the purposes of training and prediction using the proposed 1D-CNN and Bi-LSTM models.

**Figure 3:**
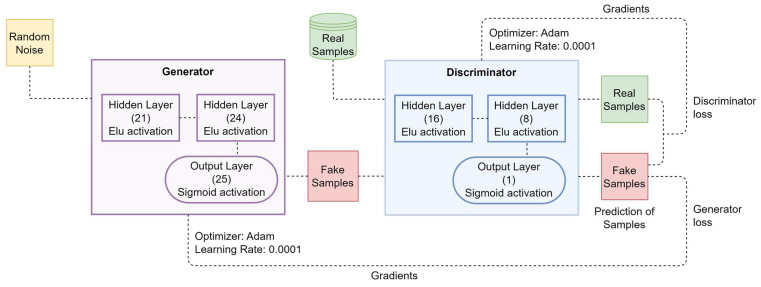
Block diagram of the proposed GAN model.

Figure 3 provides a block diagram representation of the GAN model that we constructed. In our proposed architecture, there are three distinct layers that make up the generator network. An input of random noise is provided to the first hidden layer, which consists of 21 neurons and an Elu activation function which is given by Equation (2) [34]. The subsequent hidden layer is the second one, and it consists of a total of 24 neurons and an Elu activation function. The final layer in the generator network involves an output layer that is responsible for computing the fake samples. This layer has 25 neurons and a sigmoid activation function. After the fake samples have been constructed, they are added to the real dataset and then fed into the discriminator network, which also consists of three distinct layers.

(2) $$\begin{array}{c} y=\{\begin{array}{c} \;\;x\;\;\;\;\;\;\;\;\;\;\;\;\;\;\;\;\;\;\;\;when\;\;\;\;\;\;\;\;x\;\geq 0\\ \alpha \left(e^{x}-1\right)\;\;\;\;\;\;when\;\;\;\;\;\;\;\;x<0 \end{array}\; \end{array}$$

where α is an adjustable variable which regulates the point at which the negative Elu portion reaches its saturation level.

The created fake samples, together with the actual dataset, are then fed into the discriminator network, which likewise is comprised of three distinct layers. In the first hidden layer, there are 16 neurons which are activated by an Elu activation function. Following that is the second hidden layer, which contains a total of 8 neurons and is also activated by an Elu activation function. The last output layer is made up of one neuron with a sigmoid activation function that distinguishes between real and fake data. The output of the discriminator is used to derive the loss function that the GAN model uses. Because of this, the parameters of the discriminator are updated more rapidly while the parameters of the generator are updated more slowly. Once the discriminator and generator have been trained for number of epochs, the discriminator is no longer able to differentiate between real and false data, and the generator is able to produce a new realistic dataset.

### 3.3. Dimensionality Reduction Method

The generated dataset by the GAN model contains a very large volume of patient information, making it difficult to process. This is because the information has a greater number of features, some of which belong to specific heart disease-related information while others may be an overfit for the classification process. Therefore, principal component analysis (PCA) was performed to reduce the feature set dimensionality. PCA is a dimension reduction method in which the component that has the largest projected variance is referred to as the “first principal component,” and the component that is projected to have the second largest variance is referred to as the “second principal component”, and so on [35]. By using PCA, we were able to reduce the feature set’s dimensionality of the dataset generated by the GAN model before feeding it to the 1D-CNN and Bi-LSTM predictive models. In addition, the amount of time it took to train and predict was lowered because of the reduced number of important features that were provided to the predictive models.

### 3.4. HD Diagnosis DL Models

For our 1D-CNN and Bi-LSTM predictive models, we used a tuning method to get the most accurate and relevant hyperparameter combinations possible, as tuning and determining the optimal model architecture are critical steps in the model design. We used the random search algorithm [36] to fine-tune the hyperparameters of the 1D-CNN based model, including the number of filters, filter size, learning rate, and activation function. In addition, we used the same method to fine-tune the hyperparameters of the Bi-LSTM based model, including the number of units (neurons) in each layer, the learning rate, and the activation function.

#### 3.4.1. One-Dimensional Convolutional Neural Network (1D-CNN)

The convolutional neural networks (CNNs) are the most commonly used DL models. In a CNN, the most fundamental unit consists of the input layer, the convolution layer, the activation function, and the output layer, which can be represented as shown in Equation (3) [37].

(3) $$\begin{array}{c} y=g\left(Wx+b\right) \end{array}$$

where g is an activation function; W is a convolution matrix; b is a bias; x is the input, and y is the output, respectively.

The most important component of a CNN model is the convolutional layers. Convolutional layers, which employ several various sized kernels (filters), are used for the extraction of many kinds of features from the input data [38]. Convolutional layers may be followed by a pooling layer, which is also known as the under or down sampling layer, to lower the number of extracted parameters and prevent overfitting [31]. To achieve the best training results, CNNs are trained using the backpropagation technique, with the weights constantly being changed to minimize errors [39]. CNNs can extract features from 2D input data such as photographs. However, many biological signals, including electrocardiograms (ECGs) and electroencephalograms (EEGs), only have a single dimension. This led us to construct a 1D convolution network, which refers to the application of dot products to a pre-defined window that is derived from the dataset [40].

**Figure 4:**
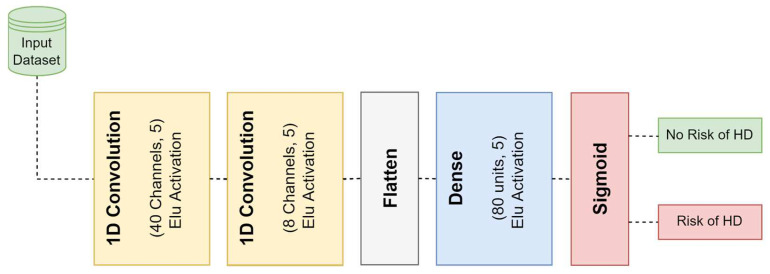
Block diagram of the proposed 1D-CNN architecture.

The four-layer deep 1D-CNN model that was constructed as part of this work is shown in Figure 4. In the first layer of the model, the input dataset is subjected to a 1D convolution, which is carried out using 40 filters with a size of (5 × 1). The activation outputs of this layer are given to the second layer, which then repeats the 1D convolution process using eight filters that each have a size of (5 × 1). On the third layer of the proposed model, there is a flattened layer. This layer is used so that the multidimensional feature vectors that are created from the second layer have their size adjusted to one that is suitable for use as an input to the layers that come after it. After the features have been obtained from the flattened layer, they are then transferred into a fully connected neural network layer that is composed of 80 different neurons. The very final layer is comprised of a sigmoid activation function layer, and its purpose is to assist in making a prediction regarding the category that the input data falls into. Table 2 provides an in-depth summary of the parameters used for all layers of the deep 4-layer 1D-CNN network that is proposed.

#### 3.4.2. Bidirectional Long Short-Term Memory (Bi-LSTM)

RNNs, also known as recurrent neural networks, are yet another modeling strategy that is frequently utilized in deep learning. In an RNN model, the output of prior states is fed into the present state, which results in a feedback loop that stores knowledge about the nodes that came before the current node [31,41]. An important benefit of RNN is its capacity to successfully learn the characteristics of information of various timesteps. However, because the initial information will eventually be forgotten, a problem with dependency over the long run may develop. As a direct consequence of this, the Long-Term Short-Term Memory (LSTM) model came into being with the ability to recall long-term dependency [41]. The architecture of LSTM is a gated structure that is made up of memory cell blocks [39]. Traditionally, the LSTM model has been used to analyze data in the forward direction while ignoring information in the future stage. Information about the future that is inputted can also provide insight into hidden states, which can aid in the process of prediction. In recent years, a new method known as the Bi-directional LSTM (Bi-LSTM) has come into existence. This method includes both forward and backward analysis of input data. Bi-LSTM is an RNN with an input layer, two dense (hidden) layers, and an output layer. The memory block that is in the hidden layers contains an input gate, forget gate, and output gate. These gates control the amount of data that is extracted as well as its features. It is up to the input gate and the forget gate to determine what new information will be stored in the cell state and what older information will be discarded. Following the completion of the cell state update, the output gate finishes off the network model’s output. In this way, the Bi-LSTM output units can automatically learn a representation that takes into consideration information from the past as well as information that will occur in the future [41].

**Figure 5:**
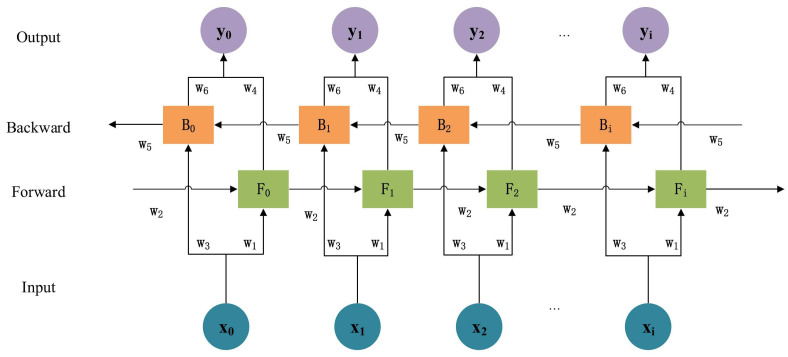
Bi-LSTM typical network [41].

As can be seen in Figure 5, a typical Bi-LSTM block is made up of four layers: an input layer, a forward layer, and a backward layer, followed by an output layer. Because of this configuration, the data can be processed in both directions by two hidden layers and then the results can be combined into a single output layer. The Bi-LSTM block, shown in Figure 5, calculates the forward hidden layer $F_{i}$, the backward hidden layer $B_{i}$, and the output layer $y_{i}$ using Equations (4)–(6) [41].

(4) $$\begin{array}{c} F_{i}=f\left(w_{1}x_{i}+w_{2}F_{i-1}+b_{i}\right) \end{array}$$

(5) $$B_{i}=f\left(w_{3}x_{i}+w_{5}B_{i+1}+b_{j}\right)$$

(6) $$y_{i}=g\left(w_{4}F_{i}+w_{6}B_{i}+b_{k}\right)$$

where, f is an update function for the state vectors of cells in the hidden layers of the Bi-LSTM block, and g is the activation function of the output layer.

**Figure 6:**
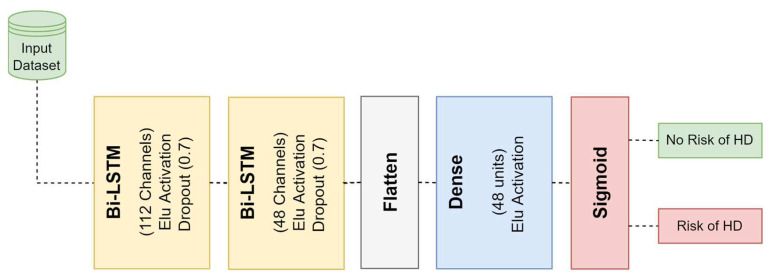
Block diagram of the proposed Bi-LSTM architecture.

Figure 6 illustrates the block diagram for our proposed 4-layer deep Bi-LSTM model. It is made up of two Bi-LSTM blocks, each of which has 112 neurons and 48 neurons in. To prevent overfitting, a dropout that is equal to 70 percent has been incorporated into each layer of the Bi-LSTM. The process of translating the multidimensional feature vectors that are produced by the second Bi-LSTM layer into a vector that is of a single dimension requires the utilization of a flattened layer as a third layer. After that, the output of the flattened layer is transferred to a fully linked layer that has a total of 48 neurons. The output predictions are computed with the help of the sigmoid activation function in the neural network’s last layer. In Table 3, the parameters that were used for each layer of the deep 4-layer Bi-LSTM network that was proposed are laid out in detail.

### 3.5. Brief Outline of Traditional ML and DL Models

For evaluating how well the proposed models can detect HD, SVM and ANN are chosen for performance comparison because they show consistent and good results on various datasets. In this part, the fundamental workings of these methods are briefly described.

#### 3.5.1. Support Vector Machine (SVM)

The input dataset is effectively split into two groups using SVM, which is an ML classification algorithm. Support vectors illustrate the line that separates the two classes so that margins can be optimized. Kernel SVM is an approach that works well with nonlinear data and can help better match the hyperplane. Currently, the SVM model makes use of four kernels, and for HD identification, the one that performs the best is chosen. They are linear kernel, sigmoid kernel, RBF kernel and polynomial kernel, accordingly [42,43].

#### 3.5.2. Artificial Neural Network (ANN)

In the field of DL, the simplest algorithms are artificial neural networks, which consist of artificial neurons serving as the network’s underlying structure. Utilizing many hidden layers is the key to developing a deep structure in an artificial neural network (ANN). Weights are used to connect artificial neurons to one another. The weighted sum is determined once the data have been transferred to the input layer. After that, the bias of each neuron is applied to the total sum of the weighted input, and an activation function is used to create an output for the neurons in following layers. To ensure that the network is properly trained, the weights are adjusted in accordance with the difference that occurs between the expected and actual results [39].

## 4. Results and Discussion

In this section, we introduce an in-depth comparison of the results achieved from implementing the 1D-CNN and Bi-LSTM models before and after utilizing the GAN model for data augmentation combined with a PCA algorithm for minimizing dataset dimensionality. In addition, a comparison of the models with the state-of-the-art methods is provided. The next subsections detail all evaluation measures with the obtained results.

### 4.1. 1D-CNN and Bi-LSTM Models Performance before Data Augmentation

The proposed 1D-CNN and Bi-LSTM architectures shown in Figure 4 and Figure 6 were implemented with the Python language and the Keras library. The Cleveland, Statlog and Comprehensive datasets (detailed in Section 3.1) were used for training and testing of these two models. To build and evaluate the 1D-CNN and Bi-LSTM models, we used the NVidia K80 GPUs available through Kaggle kernels. Both the Cleveland and Comprehensive datasets were divided into training, validation, and testing sets using a 60%:20%:20% ratio. The Statlog dataset was split into three parts, with 80% devoted to train, 10% to validation, and 10% to test. Afterwards, each part of the three datasets was standardized using the method outlined in Section 3.1. By utilizing the Adam optimizer with a (0.01) learning rate and (32) batch size, the 1D-CNN and the Bi-LSTM models were trained for 100 epochs.

**Figure 7:**
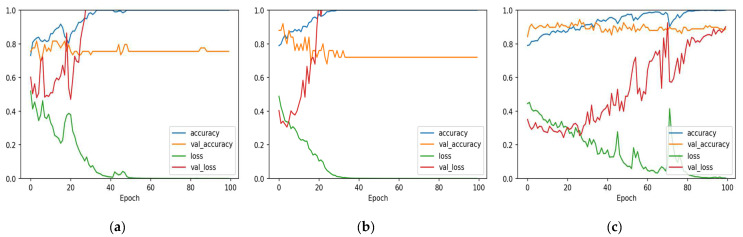
1D-CNN model accuracy and loss with epochs. (**a**) Cleveland dataset; (**b**) Statlog dataset; (**c**) Comprehensive dataset.

**Figure 8:**
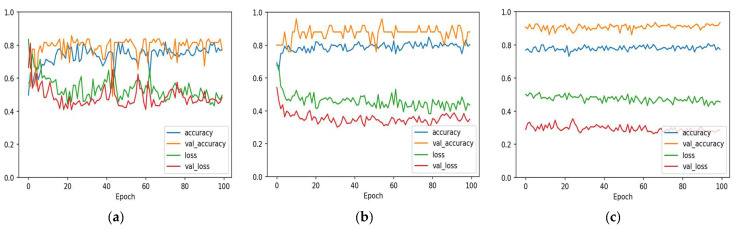
Bi-LSTM model accuracy and loss with epochs. (**a**) Cleveland dataset; (**b**) Statlog dataset; (**c**) Comprehensive dataset.

The 1D-CNN and Bi-LSTM models were trained using the training and validation sets from each of the three datasets. Accuracy and loss charts with epoch for both train and validation sets of the two models are shown in Figure 7 and Figure 8. After training for a period of time, it becomes obvious that the models are overfitting to the training data, as seen by the big gap between the accuracy and loss measures of train and validation.

To measure how well the 1D-CNN and Bi-LSTM models performed, we used the following metrics: accuracy (Acc%), specificity (Spe%), sensitivity (Sen%), F1-score (F1%), AUC chart, and confusion matrix. Following are detailed results achieved by implementing the proposed 1D-CNN and Bi-LSTM models:

#### 4.1.1. 1D-CNN Performance Evaluation

**Figure 9:**
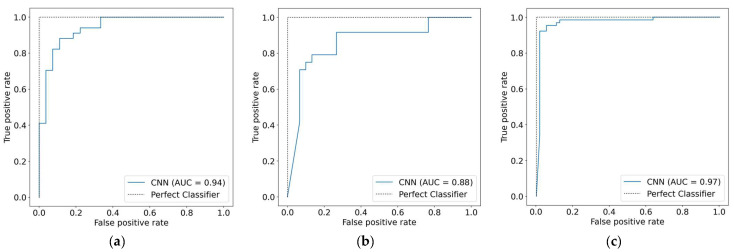
AUC measures of the 1D-CNN model. (**a**) Cleveland dataset; (**b**) Statlog dataset; (**c**) Comprehensive dataset.

**Figure 10:**
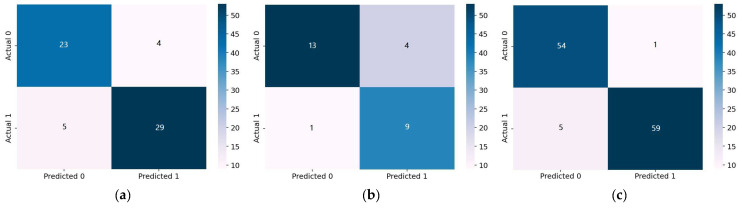
Confusion matrix of the 1D-CNN model. (**a**) Cleveland dataset; (**b**) Statlog dataset; (**c**) Comprehensive dataset.

The ability of the 1D-CNN model to detect HD was assessed using the testing set from each dataset. Table 4 shows the comparison of 1D-CNN performance on the three datasets. The model performed poorly on the Statlog dataset, with 81.48% accuracy, 81.32% specificity, 81.67% sensitivity, and 81.38% F1-score. Compared to the results obtained using the Cleveland dataset, the 1D-CNN model performed better (87.10% accuracy, 86.97% sensitivity, 86.97% specificity, and 86.97% F1-score). The best achieved results by the 1D-CNN were obtained on the Comprehensive dataset with an accuracy of 94.96%, a sensitivity of 95.18%, a specificity of 94.93%, and an F1-score of 94.95%. It is observed that the performance of the 1D-CNN model increases as the dataset increases in size. These findings provide more evidence that a larger dataset is required for the effective training of DL models. The AUC was also employed as a performance metric for further analysis. Figure 9 illustrates that 1D-CNN yielded the lowest AUC score of 0.88 when applied to the Statlog dataset, and the highest AUC score of 0.97 when applied to the Comprehensive dataset. The efficiency of the 1D-CNN model was also evaluated using the confusion matrix metric. The 1D-CNN model accurately recognized 29 of 34 HD people and 23 of 27 healthy persons in the Cleveland dataset, as shown in Figure 10a. In Figure 10b, we can see that the 1D-CNN model successfully detected 9 of 10 HD patients and 13 of 17 healthy persons when applied to the Statlog dataset. On the Comprehensive dataset, the 1D-CNN model accurately identified 59 of 64 HD people and 54 of 55 healthy persons, as shown in Figure 10c.

#### 4.1.2. Bi-LSTM Performance Evaluation

The testing set from each dataset was utilized to assess the capability of the Bi-LSTM model to detect HD. The outcomes of comparing the performance of the Bi-LSTM on the three datasets are presented in Table 5. On the Statlog dataset, the model had the worst performance, with an accuracy of 80.00%, a specificity of 79.45%, a sensitivity of 79.17%, and an F1-score of 79.28%. All of these metrics were below 80%. The Bi-LSTM model performed significantly better by utilizing the Cleveland dataset, with an accuracy of 88.52%, sensitivity of 87.80%, specificity of 89.08%, and F1-score of 88.21%. On the Comprehensive dataset, the best results that the Bi-LSTM was able to attain were an accuracy of 94.96%, a sensitivity of 95.06%, a specificity of 94.88%, and an F1-score of 94.94%. These findings provide more evidence that a larger dataset is required for the effective training of deep learning models.

**Figure 11:**
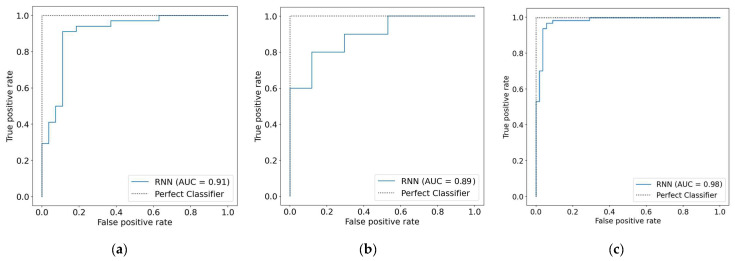
AUC measures of the Bi-LSTM model. (**a**) Cleveland dataset; (**b**) Statlog dataset; (**c**) Comprehensive dataset.

**Figure 12:**
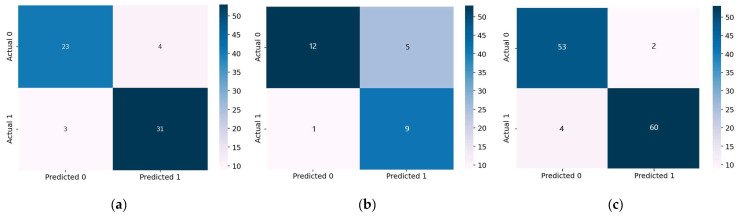
Confusion matrix of the Bi-LSTM model. (**a**) Cleveland dataset; (**b**) Statlog dataset; (**c**) Comprehensive dataset.

In addition, the AUC was utilized as a performance metric for the purpose of doing additional analysis. Figure 11 demonstrates that using Bi-LSTM to the Statlog dataset resulted in the lowest AUC score of 0.89, while applying it to the Comprehensive dataset resulted in the highest AUC value of 0.98. In addition to this, the Bi-LSTM model performance was assessed with the use of the confusion matrix measure. As shown in Figure 12a, the Bi-LSTM model correctly identified 31 of 34 participants with HD and 23 of 27 people who were healthy on the Cleveland dataset. When the Bi-LSTM model was applied to the Statlog dataset, we can see in Figure 12b that it effectively diagnosed 9 of 10 HD patients and 12 of 17 healthy participants. As shown in Figure 12c, the Bi-LSTM model correctly identified 60 of 64 participants with HD and 53 of 55 people who were healthy using the Comprehensive dataset.

### 4.2. 1D-CNN and Bi-LSTM Models Performance after Data Augmentation

Prediction accuracy was shown to be higher when the 1D-CNN and Bi-LSTM models were implemented on the larger dataset (i.e., the Comprehensive dataset) as compared to the results obtained when implementing on the smaller datasets (i.e., Cleveland and Statlog). Furthermore, implementing on the larger dataset minimized the possibility of the model overfitting to the training data. For these reasons, we developed the GAN model provided in Figure 3 to have a larger and balanced dataset for effectively training the 1D-CNN and Bi-LSTM models. The proposed GAN model architecture was built using the Python language and the Keras library. Only the Cleveland dataset was used for our GAN model development. Before building the proposed GAN model, we removed the null and noisy data from the Cleveland dataset. We then normalized the data with the scaling method described in Section 3.1. When training the generator and discriminator network of the GAN model on the Cleveland dataset, we configured the hyperparameters shown in Table 6.

After training the generator and discriminator network for 50,000 training epochs, we used the generator network to produce a new dataset of 10,000 fake samples (5000 sample with no HD and 5000 sample with HD). We then combined the new generated fake dataset with the real Cleveland dataset to train each of the developed DL models (i.e., 1D-CNN and Bi-LSTM) to diagnose HD. We combined 7764 fake samples with 236 real samples for training the DL models, and combined 1940 fake samples with 60 real samples for testing and evaluating the DL models. In total, we used 8000 samples for training and 2000 samples for testing, which represents an 80:20 split ratio for both the original and fake datasets.

**Figure 13:**
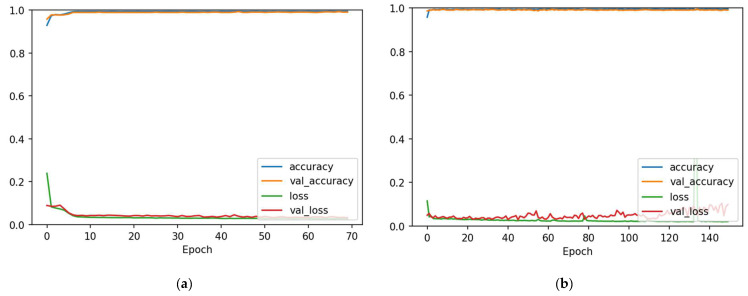
Accuracy and loss after data augmentation. (**a**) 1D-CNN model; (**b**) Bi-LSTM model.

Next, we applied the PCA method on both the train and test sets to reduce the feature set for speeding up the DL models' computation time. The 13 input features of the Cleveland dataset were reduced to only 5 features by using the PCA method. After that, both train and test datasets were normalized with the standardization method outlined in Section 3.1. We further partitioned the training set using a 90:10 split ratio into training and validation sets. This means 7200 samples were used for training, 800 samples were used for validating, and 2000 for evaluating the 1D-CNN and Bi-LSTM models. The 7200 training and 800 validation samples were then used to train the 1D-CNN for 70 epochs and Bi-LSTM for 150 epochs with a batch size equal to 32. Figure 13 displays accuracy and loss charts together with epoch for the train and validation sets of the two models. There is little variation between the accuracy and loss metrics of the train and validation sets, which indicates that the models are normally fitted to the training data.

The effectiveness of the 1D-CNN and Bi-LSTM models in detecting HD was then assessed using the 2000 testing samples. The Bi-LSTM model achieved better results as compared to the 1D-CNN model, including an accuracy of 99.3%, a specificity of 99.2%, a sensitivity of 99.3%, and an F1-score of 99.2%, as is shown in Table 7. Additionally, the prediction time for 61 samples was recorded before and after PCA was used to see the impact of feature reduction on the models’ computation time. When compared to the 1D-CNN and Bi-LSTM models that did not use PCA, the prediction time for the models that did use PCA was slightly lower.

**Figure 14:**
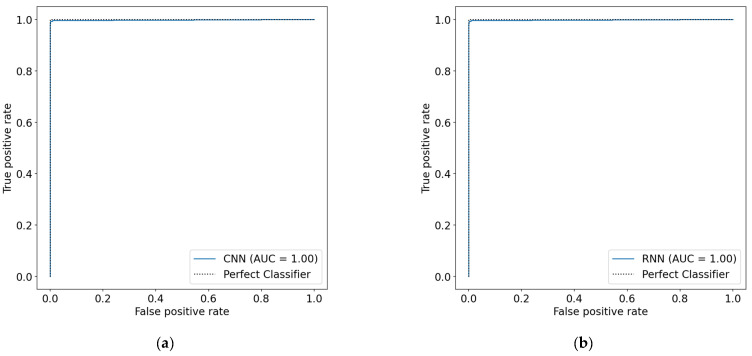
AUC measures after data augmentation. (**a**) 1D-CNN model; (**b**) Bi-LSTM model.

After applying the GAN model to augment the Cleveland dataset, the AUC measurements of both the 1D-CNN and the Bi-LSTM models improved, as shown in Figure 14. The AUC of the 1D-CNN went up to 1.00 from a previous value of 0.94, while the AUC of the Bi-LSTM increased from 0.91 to 1.00.

A comparative analysis was conducted against conventional ML and DL algorithms such as SVM and ANN to further validate the performance of the proposed 1D-CNN and Bi-LSTM models after using the Gan model to increase the dataset size. The results of this analysis are provided in Table 8. When the size of the dataset was expanded utilizing the GAN model that was presented, this comparison revealed that the 1D-CNN and Bi-LSTM models performed better than both the SVM and ANN models.

In addition, a comparison of the state-of-the-art DL models with the GAN-1D-CNN and GAN-Bi-LSTM models was carried out, and the results are presented in Table 9. This comparison was made in terms of the following three aspects: (1) the dataset on which the model is evaluated; (2) prediction accuracy; and (3) model complexity. The results of this comparison demonstrated that the proposed GAN-1D-CNN and GAN-Bi-LSTM Models performed better than other existing methods, with an accuracy of 99.10% and 99.30%, respectively.

## 5. Conclusions

The issue of HD diagnosis has been addressed in this study by making use of various patient’s physiological data as input information. These patient’s physiological data included fasting blood sugar, heart rate, (electrocardiogram) ECG, blood pressure, cholesterol level, and so on. There have been two different DL-based HD diagnostic frameworks suggested, GAN-1D-CNN and GAN-Bi-LSTM. These frameworks are comprised of two basic components: (1) a generative adversarial network (GAN) model; and (2) either a one-dimensional convolutional neural network (1D-CNN) model or a bidirectional long short-term memory (Bi-LSTM) model. The GAN model has been utilized to produce additional fake samples with the intention of enhancing the limited and unbalanced dataset that is currently available to better train the proposed 1D-CNN and Bi-LSTM HD prediction models. This was accomplished via the usage of the GAN model. Both proposed HD diagnostic models, namely GAN-1D-CNN and GAN-Bi-LSTM, are tested on three different unbalanced datasets, including Cleveland, Statlog, and Comprehensive. To conduct performance analysis for the GAN-1D-CNN and GAN-Bi-LSTM frameworks, many measures, such as accuracy, specificity, sensitivity, F1-score, area under the curve (AUC), and confusion matrix, were utilized. The performance of the Bi-LSTM model was improved by using the GAN model on the Cleveland dataset. As a result, the model’s accuracy increased to 99.3%, its specificity increased to 99.2%, its sensitivity increased to 99.3%, and its F1 score increased to 99.2%. A similar impact was seen on the performance of the 1D-CNN model, which ended up with an accuracy score of 99.1%, a specificity score of 99.1%, a sensitivity score of 99.1%, and an F1 score of 99.1%. In addition, the prediction time for the 1D-CNN and Bi-LSTM models was improved by the utilization of the PCA approach. It decreased from 72.6 milliseconds to 68.8 milliseconds for the 1D-CNN model, while it decreased from 80.4 milliseconds to 74.8 milliseconds for the Bi-LSTM model. Finally, both GAN-1D-CNN and GAN-Bi-LSTM were comparatively analyzed with various existing ML and DL methods (i.e., SVM and ANN) and other state-of-the-art works. The comparison results showed that the proposed DL-based HD diagnostic frameworks performed better than their competitors. In the future, we will attempt to implement the proposed DL models of this paper on cloud computing platforms and apply alternative DL models to identify cardiac disease quickly and accurately.

## Figures and Tables

**Table 1:** Attributes description of Cleveland and Statlog datasets [25,26].

Attribute	Type	Summary
Age	Number	The number of years
Sex.	Category	0: Female or 1: male
Cp.	Category	Chest pain of a specific type (1: typical angina, 2: atypical angina, 3: non anginal pain, 4: asymptomatic)
Trestbps	Number	Blood pressure at rest measured in mmHg
Chol	Number	Cholesterol level measured in mg/dL
Fbs	Category	Fasting glucose level over 120 mg/dL (0: false, 1: true)
Restecg.	Category	Electrocardiogram reading in resting state (0: normal, 1: ST-T wave abnormalities, and 2: left ventricular hypertrophy)
Thalach	Number	Results of a thallium stress test showing the highest possible heart rate
Exang.	Category	Angina during exercise (1 indicates yes, 0 indicates no)
Oldpeak	Number	Exercise ST depression versus rest
Slope	Category	ST segment inclination during exercise (1: up, 2: flat, 3: down)
Ca.	Category	Significant fluoroscopically colored vessel number
Thal	Category	Test results for thulium stress: 3: normal, 6: a fixed defect, and 7: a reversible defect
Num	Category	HD status (0 indicates less than 50% diameter narrowing, 1indicates more than 50% diameter narrowing)

**Table 2:** A summary table of the proposed 1D-CNN model.

Layer	Type	No. of Filters	Kernel Size	Activation Function
1	1D-Convolution	40	5	Elu
2	1D-Convolution	8	5	Elu
3	Flatten	-	-	-
4	Fully Connected	80	-	Elu
5	Fully Connected	1	-	Sigmoid

**Table 3:** A summary table of the proposed Bi-LSTM model.

Layer	Type	No. of Neurons	Dropout Ratio	Function
1	Bi-LSTM	112	0.7	Elu
2	Bi-LSTM	48	0.7	Elu
3	Flatten	-	-	-
4	Fully Connected	48	-	Elu
5	Fully Connected	1	-	Sigmoid

**Table 4:** The 1D-CNN model performance analysis.

Dataset	Records	Features	Accuracy	Specificity	Sensitivity	F1-Score
Cleveland	303	14	87.10%	86.97%	86.97%	86.97%
Statlog	270	14	81.48%	81.32%	81.67%	81.38%
Comprehensive	1190	12	94.96%	94.93%	95.18%	94.95%

**Table 5:** The Bi-LSTM model performance analysis.

Dataset	Records	Features	Accuracy	Specificity	Sensitivity	F1-Score
Cleveland	303	14	88.52%	89.08%	87.80%	88.21%
Statlog	270	14	80.00%	79.45%	79.17%	79.28%
Comprehensive	1190	12	94.96%	94.88%	95.06%	94.94%

**Table 6:** Hyperparameter configuration of the proposed GAN model.

Hyperparameter	Value	Description
Epoch	50,000	Number of training iterations
Batch size	32	Number of batch samples per iteration
Learning rate	0.0001	Learning rate
Optimizer	Adam	Optimization algorithm

**Table 7:** Performance comparison before and after using the GAN model on the Cleveland dataset.

	Before Augmentation and PCA					After Augmentation and PCA				
Method	Acc (%)	Spe (%)	Sen (%)	F1 (%)	Prediction Time (ms)	Acc (%)	Spe (%)	Sen (%)	F1 (%)	Prediction Time (ms)
1D-CNN	87.10	86.97	86.97	86.97	72.6	99.1	99.1	99.1	99.1	68.8
Bi-LSTM	88.52	89.08	87.80	88.21	80.4	99.3	99.2	99.3	99.2	74.8

**Table 8:** Benchmarking the proposed models against ML and DL models for the Cleveland dataset.

Method	Accuracy	Precision	Recall	F1-Score
SVM	86.88%	87.76%	85.95%	86.44%
ANN	93.44%	93.83%	92.97%	93.30%
Proposed GAN-CNN	99.1%	99.1%	99.1%	99.1%
Proposed GAN-Bi-LSTM	99.3%	99.2%	99.3%	99.2%

**Table 9:** Comparison of GAN-1D-CNN and GAN-Bi-LSTM models with State-Of-The-Art.

Study	Method	Accuracy	Dataset	Model Complexity
[18]	DNN	94.2%	Cleveland	6 layers (3 Dense, 2 Dropout, 1 Output)
[21]	SAE-ANN ^1^	90%	Framingham	SAE network (5 layers)
[20]	ANN	92%	Cleveland	2 layers (1 Dense, 1 Output)
[22]	CNN	94.78%	Cleveland	5 layers (2 Conv1D, 2 Dropout, 1 Output)
[23]	RNN-GRU ^2^	98.4%	Cleveland	7 layers of GRUs
[24]	OCI-DBN ^3^	94.61%	Cleveland	3 layers (2 Dense, 1 Output)
[19]	DNN	93.33%	Cleveland	3 layers (2 Dense, 1 Output)
**Proposed**	**GAN-1D-CNN** **GAN-Bi-LSTM**	**99.10%** **99.30%**	**Cleveland**	**4 layers (2 Conv1D, 1 Dense, 1 Output)** **4 layers (2 Bi-LSTM, 1 Dense, 1 Output)**

^1^ SAE: sparse autoencoder; ^2^ GRU: Gated Recurrent Unit; ^3^ OCI-DBN: Optimally Configured and Improved Deep Belief Network.

## Data Availability

The machine-learning repository at UCI provides access to the datasets that were used in this study.

## References

[1] Alnemari W., Bakry S., Albagami S., AL-Zahrani S., Almousa A., Alsufyani A., Siddiqui M. (2022). Awareness and knowledge of Rheumatic heart disease among medical students comparing to other health specialties students in Umm Al-Qura University, Makkah city, KSA-Analytic cross-sectional study. Med. Sci..

[2] Elhoseny M., Mohammed M.A., Mostafa S.A., Abdulkareem K.H., Maashi M.S., Garcia-Zapirain B., Mutlag A.A., Maashi M.S. (2021). A new multi-agent feature wrapper machine learning approach for heart disease diagnosis. Comput. Mater. Contin..

[3] Sarra R.R., Dinar A.M., Mohammed M.A. (2022). Enhanced accuracy for heart disease prediction using artificial neural network. Indones. J. Electr. Eng. Comput. Sci..

[4] Odah M.M., Alfakieh H.O., Almathami A.A., Almuashi I.M., Awad M., Ewis A.A. (2022). Public Awareness of Coronary Artery Disease and its Risk Factors Among Al-Qunfudah Governorate Population. J. Umm Al-Qura Univ. Med. Sci..

[5] Sharma M. (2019). ECG and Medical Diagnosis Based Recognition & Prediction of Cardiac Disease Using Deep Learning. J. Sci. Technol. Res..

[6] Mhamdi L., Dammak O., Cottin F., Dhaou I.B. (2022). Artificial Intelligence for Cardiac Diseases Diagnosis and Prediction Using ECG Images on Embedded Systems. Biomedicines.

[7] Sarra R.R., Dinar A.M., Mohammed M.A., Abdulkareem K.H. (2022). Enhanced Heart Disease Prediction Based on Machine Learning and χ2 Statistical Optimal Feature Selection Model. Designs.

[8] Rahman A.U., Saeed M., Mohammed M.A., Jaber M.M., Garcia-Zapirain B. (2022). A novel fuzzy parameterized fuzzy hypersoft set and riesz summability approach based decision support system for diagnosis of heart diseases. Diagnostics.

[9] Bizopoulos P., Koutsouris D. (2019). Deep Learning in Cardiology. IEEE Rev. Biomed. Eng..

[10] Rahman A.U., Saeed M., Mohammed M.A., Krishnamoorthy S., Kadry S., Eid F. (2022). An Integrated Algorithmic MADM Approach for Heart Diseases’ Diagnosis Based on Neutrosophic Hypersoft Set with Possibility Degree-Based Setting. Life.

[11] Nasser A.R., Hasan A.M., Humaidi A.J., Alkhayyat A., Alzubaidi L., Fadhel M.A., Santamaría J., Duan Y. (2021). IoT and Cloud Computing in Health-Care: A New Wearable Device and Cloud-Based Deep Learning Algorithm for Monitoring of Diabetes. Electronics.

[12] Mathur P., Srivastava S., Xu X., Mehta J.L. (2020). Artificial Intelligence, Machine Learning, and Cardiovascular Disease. Clin. Med. Insights. Cardiol..

[13] Ontor M.Z.H., Ali M.M., Ahmed K., Bui F.M., Al-Zahrani F.A., Mahmud S.M.H., Azam S. (2022). Early-stage cervical cancerous cell detection from cervix images using yolov5. Comput. Mater. Contin..

[14] Subahi A.F., Khalaf O.I., Alotaibi Y., Natarajan R., Mahadev N., Ramesh T. (2022). Modified Self-Adaptive Bayesian Algorithm for Smart Heart Disease Prediction in IoT System. Sustainability.

[15] Sandhiya S., Palani U. (2020). An effective disease prediction system using incremental feature selection and temporal convolutional neural network. J. Ambient. Intell. Humaniz. Comput..

[16] Zamzmi G., Hsu L.Y., Li W., Sachdev V., Antani S. (2021). Harnessing Machine Intelligence in Automatic Echocardiogram Analysis: Current Status, Limitations, and Future Directions. IEEE Rev. Biomed. Eng..

[17] Bharti R., Khamparia A., Shabaz M., Dhiman G., Pande S., Singh P. (2021). Prediction of Heart Disease Using a Combination of Machine Learning and Deep Learning. Comput. Intell. Neurosci..

[18] Ali L., Rahman A., Khan A., Zhou M., Javeed A., Khan J.A. (2019). An Automated Diagnostic System for Heart Disease Prediction Based on X^2^ Statistical Model and Optimally Configured Deep Neural Network. IEEE Access.

[19] Das S., Sharma R., Gourisaria M.K., Rautaray S.S., Pandey M. (2020). Heart Disease Detection Using Core Machine Learning and Deep Learning Techniques: A Comparative Study. Int. J. Emerg. Technol..

[20] Mienye I.D., Sun Y., Wang Z. (2020). Improved sparse autoencoder based artificial neural network approach for prediction of heart disease. Inform. Med. Unlocked.

[21] Sajja T.K., Kalluri H.K. (2020). A deep learning method for prediction of cardiovascular disease using convolutional neural network. Rev. D' Intell. Artif..

[22] Krishnan S., Magalingam P., Ibrahim R.B. (2020). Advanced Recurrent Neural Network with Tensorflow for Heart Disease Prediction. Int. J. Adv. Sci..

[23] Ali S.A., Raza B., Malik A.K., Shahid A.R., Faheem M., Alquhayz H., Kumar Y.J. (2020). An optimally configured and improved deep belief network (OCI-DBN) approach for heart disease prediction based on Ruzzo–Tompa and stacked genetic algorithm. IEEE Access.

[24] Janosi A., Steinbrunn W., Pfisterer M., Detrano R. UCI Machine Learning Repository: Heart Disease Dataset. https://archive-beta.ics.uci.edu/ml/datasets/heart+disease.

[25] UCI Machine Learning Repository: Statlog (Heart). https://archive-beta.ics.uci.edu/ml/datasets/statlog+heart.

[26] Vijayashree J., Parveen Sultana H. (2020). Heart disease classification using hybridized Ruzzo-Tompa memetic based deep trained Neocognitron neural network. Health Technol..

[27] Heart Disease Dataset (Comprehensive). https://www.kaggle.com/datasets/sid321axn/heart-statlog-cleveland-hungary-final.

[28] Wang J., Liu X., Wang F., Zheng L., Gao F., Zhang H., Zhang X., Xie W., Wang B. (2021). Automated interpretation of congenital heart disease from multi-view echocardiograms. Med. Image Anal..

[29] Baghel N., Dutta M.K., Burget R. (2020). Automatic diagnosis of multiple cardiac diseases from PCG signals using convolutional neural network. Comput. Methods Programs Biomed..

[30] Luo X., Yang L., Cai H., Tang R., Chen Y., Li W. (2021). Multi-classification of arrhythmias using a HCRNet on imbalanced ECG datasets. Comput. Methods Programs Biomed..

[31] Chandra V., Singh V., Sarkar P.G. (2020). A Survey on the Role of Deep Learning in 2D Transthoracic Echocardiography. Int. J. Sci. Technol. Res..

[32] Zhang H. (2020). Heartbeat monitoring with an mm-wave radar based on deep learning: A novel approach for training and classifying heterogeneous signals. Remote Sens. Lett..

[33] Olaniyi E.O., Oyedotun O.K., Adnan K. (2015). Heart Diseases Diagnosis Using Neural Networks Arbitration. Int. J. Intell. Syst. Appl..

[34] Amarbayasgalan T., Park K.H., Lee J.Y., Ryu K.H. (2019). Reconstruction error based deep neural networks for coronary heart disease risk prediction. PLoS ONE.

[35] Samir A.A., Rashwan A.R., Sallam K.M., Chakrabortty R.K., Ryan M.J., Abohany A.A. (2021). Evolutionary algorithm-based convolutional neural network for predicting heart diseases. Comput. Ind. Eng..

[36] Bergstra J., Bengio Y. (2012). Random search for hyper-parameter optimization. J. Mach. Learn Res..

[37] Li Z., Zhou D., Wan L., Li J., Mou W. (2020). Heartbeat classification using deep residual convolutional neural network from 2-lead electrocardiogram. J. Electrocardiol..

[38] Lih O.S., Jahmunah V., San T.R., Ciaccio E.J., Yamakawa T., Tanabe M., Kobayashi M., Faust O., Acharya U.R. (2020). Comprehensive electrocardiographic diagnosis based on deep learning. Artif. Intell. Med..

[39] Alkhodari M., Fraiwan L. (2021). Convolutional and recurrent neural networks for the detection of valvular heart diseases in phonocardiogram recordings. Comput. Methods Programs Biomed..

[40] Dang H., Sun M., Zhang G., Qi X., Zhou X., Chang Q. (2019). A Novel Deep Arrhythmia-Diagnosis Network for Atrial Fibrillation Classification Using Electrocardiogram Signals. IEEE Access.

[41] Guo C., Zhang J., Liu Y., Xie Y., Han Z., Yu J. (2020). Recursion Enhanced Random Forest with an Improved Linear Model (RERF-ILM) for Heart Disease Detection on the Internet of Medical Things Platform. IEEE Access.

[42] Pedregosa F., Varoquaux G., Gramfort A., Michel V., Thirion B., Grisel O., Blondel M., Prettenhofer P., Weiss R., Dubourg V. (2011). Scikit-learn: Machine learning in Python. J. Mach. Learn. Res..

[43] Mastoi Q.U., Wah T.Y., Mohammed M.A., Iqbal U., Kadry S., Majumdar A., Thinnukool O. (2022). Novel DERMA Fusion Technique for ECG Heartbeat Classification. Life.

